# Apgar scores in puppies following the induction of etomidate compared with alfaxalone or propofol for cesarean section

**DOI:** 10.14202/vetworld.2024.527-534

**Published:** 2024-03-05

**Authors:** Thanikul Srithunyarat, Supranee Jitpean, Piyasak Wipoosak, Chalermkwan Nonthakotr, Nitaya Boonbal, Panisara Kunkitti, Suvaluk Seesupa

**Affiliations:** 1 Division of Surgery, Faculty of Veterinary Medicine, Khon Kaen University, 40002, Thailand; 2 Veterinary Teaching Hospital, Faculty of Veterinary Medicine, Khon Kaen University, 40002, Thailand; 3 Division of Theriogenology, Faculty of Veterinary Medicine, Khon Kaen University, 40002, Thailand

**Keywords:** anesthesia, Apgar, puppies, viability, vigorous

## Abstract

**Background and Aim:**

The Apgar score is a useful assessment of neonatal viability in dogs. The Apgar score in puppies born by cesarean section can be lower than vaginal delivery because all anesthetic drugs can cross the placenta. Therefore, anesthetic drugs with minimal cardiorespiratory effect and rapid elimination are recommended for cesarean section. The present study aimed to compare Apgar scores in puppies born after the induction of etomidate, alfaxalone or propofol, and those maintained with isoflurane inhalation during cesarean section.

**Materials and Methods:**

Thirty-six bitches were equally divided in the three anesthetic drug groups. Modified Apgar scores were assessed at 5, 15, and 60 min after delivery. Intraoperative vital signs and Apgar scores were compared using a linear mixed model and adjusted pairwise comparisons using Bonferroni analysis.

**Results:**

A total of 125 puppies were included in this study. Age, body weight, litter size, type of surgery, delivery time, anesthetic and surgical duration, and intraoperative vital signs did not significantly differ between the groups. Puppies in the alfaxalone and propofol groups had significantly higher Apgar scores than the etomidate group in both elective and emergency surgery. In elective surgery, Apgar scores at 5 min after delivery did not differ significantly between groups. At 15 and 60 min after delivery, Apgar scores in the etomidate group were significantly lower than those in the other groups. In emergency surgery, Apgar scores were significantly lower in the etomidate group than in the alfaxalone group at all time points.

**Conclusion:**

Induction with alfaxalone and propofol resulted in better outcomes with higher Apgar scores and neonatal viability than etomidate. Therefore, alfaxalone and propofol should be used as anesthetic induction drugs in both elective and emergency cesarean sections.

## Introduction

Dystocia is a common problem in canine pregnancy that affects neonatal survival. The prevalence of canine dystocia is 3.7%, and 60%–80% of dystocia bitches require surgical intervention [1]. The neonatal mortality rate after cesarean section is as high as 8%–19.6% [2]. Because fetal asphyxia is the main cause of fetal death, the beginning of respiration is crucial and has a long-term effect on neonatal viability [3]. Therefore, neonatal assessment and assistance are imperative for improving neonatal survival in dogs [4]. There are various methods for assessing canine neonatal viability, including measurement of umbilical lactate, blood glucose, blood gas analysis, and Apgar score [4–10]. Although these blood analyses, especially the umbilical lactate concentration, are useful for predicting neonatal mortality, they are invasive and difficult to perform in puppies [6]. The Apgar score has been used in human neonatal assessment and has later been modified for use in dogs [4, 5]. The Apgar score is noninvasive, simple, inexpensive, practical, reliable, repeatable, and correlated with umbilical lactate concentration; therefore, it is recommended for assessing and predicting canine neonatal viability [5, 6, 11]. The Apgar score can also be used to compare the effects of anesthetic drugs on canine neonatal viability, and several studies have used it to compare anesthetic protocols within 60 min after delivery [9, 12–14]. A low Apgar score indicates a severely distressed puppy, and prompt resuscitation is warranted to increase the chances of neonatal survival [4, 5].

Cesarean section is a common surgical procedure. Fetal distress with a fetal heart rate of less than 180 beats/min (bpm) is the most common indication for emergency cesarean section [15]. Several factors affect neonatal survival, including emergency surgery, brachycephalic breeds, litter size, body size, spontaneous breath and vocalization, and anesthetic procedure [2, 16, 17]. The two most common causes of fetal depression following cesarean section are fetal hypoxia and anesthetic effects [7]. The anesthetic protocol is one of the important factors affecting puppy’s viability because all anesthetic drugs can cross the blood-placental barrier to the fetus. Although distribution and redistribution allow the transfer of the drug back to the bitch, elimination of the remaining concentration in the fetus depends on the effects of the drug and the function of fetal liver and kidneys. Anesthetic drugs, duration of anesthesia, and analgesia affect maternal and neonatal outcomes in dogs [2, 12, 13, 16]. Anesthetic drugs with minimal cardiorespiratory effect and rapid elimination, followed by isoflurane or sevoflurane maintenance, are recommended as general anesthetic protocols in canine cesarean section [2]. Propofol and alfaxalone are recommended anesthetic induction drugs for general anesthesia in cesarean section, and alfaxalone is associated with better Apgar scores than propofol [12, 13]. Etomidate is an anesthetic induction drug with minimal effects on the cardiovascular and respiratory systems and has been used in dogs with cardiovascular or systems complications. However, no study has reported the effect of etomidate induction on Apgar scores in puppies after cesarean delivery.

The present study aimed to compare Apgar scores in puppies born after the induction of etomidate, alfaxalone, or propofol and those maintained with isoflurane inhalation during cesarean section.

## Materials and Methods

### Ethical approval and Informed consent

This study was approved by the Ethics Committee of KKU (IACUC-KKU-62/62). All owners were informed and provided their written consent before participation.

### Study period and location

This prospective clinical study was conducted from August 2019 to December 2021. All dogs were admitted to Khon Kaen University (KKU) Veterinary Teaching Hospital (VTH), Khon Kaen, Thailand, for treatment according to the routine protocol.

### Animals

All dogs included in the study were client-owned and were admitted to VTH, KKU, according to the criteria for elective or emergency cesarean section. Dogs admitted for elective cesarean section were withheld food for 6 h before surgery, whereas emergency dogs were withheld food from the time of admission. History taking, physical examination, blood tests (hematology, creatinine, alanine aminotransferase, total protein, albumin, and blood parasite screening), and imaging diagnosis (abdominal radiography and/or ultrasonography) were routinely performed in all dogs. The gestational age was estimated by ultrasonographic measurement of the fetal biparietal diameter, history, and conception time. Dogs with blood parasites (e.g., *Ehrlichiosis* and *Anaplasmosis*), abnormal blood chemistry test results, or uterine infections were excluded from the study.

### Anesthesia

Before surgery, the dogs were stabilized using 5–10 mL/kg/h intravenous Lactate Ringer’s solution (Lactate Ringer’s solution^®^, General Hospital Products Public Co Ltd., Thailand). Hair was clipped, and the skin was preliminarily scrubbed with chlorhexidine soap. Preoxygenation for at least 10 min was administered to all dogs before induction. Cefazolin (Cefaben^®^, L.B.S. Laboratory Ltd., Thailand), 25 mg/kg, was administered intravenously before induction or at least 15 min before surgery. No sedative drug was administered. The dogs were randomly allocated into three groups according to the anesthetic induction drugs, and the Apgar examiner and board-certified surgeon were blinded. Dogs were anesthetized with etomidate (Etomidate-Lipuro, B Braun, Germany), alfaxalone (Alfaxan^®^, Jurox, Australia), or propofol (Profol™, Baxter, India) titrated dose until endotracheal intubation was allowed, and isoflurane inhalation with pure oxygen was used to maintain the surgical anesthetic stage. During induction, smoothness was scored by the same veterinarian. Smooth scores were graded as follows: 0 was poor; 1 was fair; 2 was good; and 3 was excellent. Five μg/kg of fentanyl (Fentanyl-Hameln, Siam Bioscience, Thailand) was intravenously administered after delivery. Lidocaine (Lidocaine Hydrochloride, the Government Pharmaceutical Organization, Thailand), 4 mg/kg diluted with normal saline to reach 0.6 mL/kg, was instilled into the intraperitoneal region after all puppies were delivered. Morphine (Morphine Sulfate Injection, M&H Manufacturing Co. Ltd., Thailand), 0.5 mg/kg, was subcutaneously administered after surgery or 30 min after fentanyl injection. Cephalexin (Sporicef^®^, M&H Manufacturing Co. Ltd., Thailand), 20–30 mg/kg, twice daily for 7 days, and tramadol (Tradolgesic^®^, Bangkok Lab and Cosmetic Co., Ltd., Thailand), 4–6 mg/kg, twice daily or as needed for 3 days, were given as oral home medicines. The anesthetic induction drug, induction time, time of puppy delivery, anesthetic duration, heart rate, respiratory rate, blood pressure, and isoflurane concentration were recorded. All dogs were anesthetized by the same board-certified anesthetist.

### Surgery

Elective cesarean section was performed in dogs with a history of dystocia, previous cesarean section, or brachycephalic breeds. The gestational age was between 60 and 63 days. Emergency cesarean section was performed in dogs with dystocia or in cases of fetal distress in which the fetal heart rate was less than 180 bpm. The dogs were aseptically prepared in the oblique dorsal recumbency position. Caudal midline laparotomy was performed through the linea alba according to the standard surgical method [18]. The uterus was handled, and the uterine body or horn was incised. All puppies were delivered, and neonatal care was provided by veterinary assistants. Oxytocin (5 IU) was further intravenously administered. The seromuscular layer of the uterus was closed with a two-layer closure using 3/0 polyglactin 910 (Ethicon Coated Vicryl, Johnson and Johnson, USA) using the right-angle Cushing and Lambert pattern. After delivery, dogs with spayed cesarean section, ovarian pedicles, and uterine vessels were double ligated with 2/0–3/0 polyglactin 910, and the uterine stump end was closed using a Parker–Kerr pattern with 3/0 polyglactin 910. The omentum was placed over the surgical area, and the abdominal wall was closed routinely with a simple interrupted pattern using 2/0–3/0 polyglactin 910 at the linea alba and subcutaneous layer and 2/0–3/0 pseudo-monofilament polyamide (Supramid, Serag Wiessner, Germany) at the skin. After full recovery, the dogs were discharged home. All procedures were performed by the same board-certified surgeon.

### Neonatal care

The amnion sac was removed, oronasal cavity and pharynx were cleared, and the body was dried using dry towels. The umbilical cord was ligated and cut approximately 2 cm from the abdominal wall using 3/0 silk (Silk, Unik Surgical Suture, Taiwan). After preliminary neonatal care, a physical examination was performed on all puppies for gender, birth weight, and congenital deformities such as cleft palate, omphalocele, and atresia ani. All puppies were transferred to an oxygen cage with controlled temperature (32°C–33°C) and humidity (40%) and then introduced to their bitches after recovery. Each puppy was cared for individually by a veterinary nurse. Puppies with congenital defects were excluded from this study.

### Apgar score

The time of fetal traction from the uterus was used as the birth time, and immediate neonatal care was performed. Modified Apgar scores at 5, 15, and 60 min after birth were used to score Apgar [4, 5]. Five Apgar parameters were evaluated, including heart rate, mucus color, reflex irritability, motility, and respiratory effort. Heart rate >220 bpm was rated as 2, between 180 and 220 bpm was rated as 1, and <180 bpm was rated as 0. Pink mucus color was rated as 2, pale as 1, and cyanosis as 0. Reflex irritability was assessed by compression at the tip of a paw; vigorous reflex was scored as 2, grimace as 1, and absence of reflex irritability as 0. Being active with strong motility was scored as 2, some movement was scored as 1, and flaccidity was scored as 0 in the motility parameter. Respiratory rate >15 breaths/min was rated as 2, between 6 and 15 breaths/min was rated as 1, and <6 breaths/min was rated as 0. The total Apgar scores were classified into three classes for viability prediction: Low, moderate, and high (0–3, 4–6, and 7–10, respectively) [4, 5]. All puppies were examined by the same veterinarian.

### Statistical analysis

Data of bitches and puppies represented as continuous variables were evaluated for normality using the Shapiro–Wilk test. To explore the homogeneity of baseline data, bitch data in continuous variables (age, weight, delivery time, anesthetic time, and surgical time) were compared between the intervention groups (anesthetic drug) using one-way analysis of variance with Bonferroni adjustment. Comparisons of discrete data (litter number, litter size, and number of puppies born following cesarean section) between groups were calculated using the Kruskal–Wallis test with Dunn’s test for multiple comparisons. The number of bitches in each type of surgery (elective, emergency, and spayed cesarean section) was analyzed using the Chi-square test with Pearson’s correlation coefficient. The effect of intervention on intraoperative vital signs was analyzed using a linear mixed model, with the anesthetic group as a fixed effect and the dog as a random effect. Age, weight, breed, and litter number were added as covariates to the model. Pairwise comparisons were adjusted for multiplicity using the Bonferroni test.

To test the effect of the puppy characteristics data in the anesthetic group and the time point on the Apgar score, a linear mixed model and random slope on time (repeated) with a covariate structure (unstructured) was used. If the effect of interaction between the anesthetic group and time was significant, Bonferroni was used to adjust pairwise comparisons (between groups in each time point). All analyses were performed using the STATA software (Stata 18, StataCorp LLC, USA). Analysis with p < 0.05 was interpreted as statistically significant.

## Results

### The bitches

Thirty-six bitches were equally divided in the three anesthetic drug groups. The bitch breeds included in this study were French Bulldog (n = 13), Chihuahua (n = 4), Beagle (n = 2), Bully (n = 2), Poodle (n = 2), Jack Russel (n = 2), Pomeranian (n = 1), Bulldog (n = 1), Golden Retriever (n = 1), Siberian Husky (n = 2), Dachshund (n = 1), Corgi (n = 2), and Mixed breed (n = 3). The French Bulldog, Chihuahua, and Bulldog were classified as brachycephalic breeds and were allocated to each group. Body weight, age, litter number, litter size, number of puppies born following cesarean section, and ultrasound fetal heart rate did not differ significantly between the groups (Table-1). The litter number ranged from one to four and the litter size ranged from one to eleven. Six dogs underwent emergency cesarean section and six underwent elective surgery in each group. The average gestation period was 61.5 days in the bitches. Six patients in the etomidate group, three in the alfaxalone group, and seven in the propofol group underwent spayed cesarean section. No significant differences were observed between the anesthetic groups in the number of dogs in each type of surgery, the time of delivery, anesthesia, and surgery (Table-2). The anesthetic induction dosages of etomidate, alfaxalone, and propofol were 1.98 ± 0.33, 2.03 ± 0.12, and 5.44 ± 0.64 mg/kg, respectively.

**Table-1:** Descriptive data of the dogs in each group.

Parameters	Etomidate (n = 12)	Alfaxalone (n = 12)	Propofol (n = 12)	p-value
Body weight (kg)	13.58 ± 9.76	13.66 ± 5.55	14.63 ± 6.89	0.93
Age (year)	2.48 ± 2.07	2.83 ± 2.96	2.05 ± 1.26	0.69
Litter number	1.36 ± 0.5	1.67 ± 0.78	1.5 ± 1.00	0.64
Litter size	4.00 ± 2.98	3.92 ± 2.15	4.50 ± 1.73	0.31
Number of cesarean puppies	3.42 ± 2.78	3.58 ± 2.23	3.75 ± 2.09	0.75
Fetal heart rate (beats per min)	199.25 ± 34.59	191.58 ± 31.76	172.58 ± 38.11	0.17

The data are represented as mean ± standard deviation

**Table-2:** Delivery time, anesthetic time, and surgical time in each group.

Time	Etomidate (n = 12)	Alfaxalone (n = 12)	Propofol (n = 12)	p-value
Delivery time (min)	6.08 ± 2.71	5.75 ± 1.86	6.17 ± 2.25	0.89
Anesthetic time (min)	34.58 ± 11.20	33.00 ± 6.52	36.17 ± 11.27	0.74
Surgical time (min)	32.92 ± 11.39	32.67 ± 6.76	33.92 ± 11.60	0.95

Data are represented as mean ± standard deviation, Delivery time: Time from induction to last puppy delivery, Anesthetic time: Time from induction to ending isoflurane, Surgical time: Time from incision to finishing the operation

Anesthetic smooth scores differed significantly between the groups. Etomidate smooth scores were lower than those of propofol (Table-3). The mean intraoperative heart rate in the etomidate group was significantly lower than that in the propofol group, but not in the alfaxalone group. There were no significant differences in the mean intraoperative vital signs of respiratory rate, systolic blood pressure, mean blood pressure, SpO_2_, and isoflurane concentration between the groups (Table-3).

**Table-3:** Intraoperative parameters of the dogs during cesarean section.

Parameters	Etomidate (n = 12)	Alfaxalone (n = 12)	Propofol (n = 12)	p-value
Smooth score (/3)	2.25 ± 0.71^a^	2.61 ± 0.78^ab^	2.89 ± 0.22^b^	0.04*
Heart rate (bpm)	116.18 ± 9.40^a^	124.65 ± 14.47^ab^	130.47 ± 12.24^b^	0.01*
Respiratory rate (bpm)	20.75 ± 12.29	19.08 ± 14.74	14.67 ± 8.46	0.45
Systolic (mmHg)	116.25 ± 14.18	112.32 ± 11.51	115.92 ± 11.58	0.65
Mean (mmHg)	82.61 ± 11.58	75.55 ± 10.27	82.83 ± 10.31	0.12
SpO_2_ (%)	98.58 ± 1.24	98.33 ± 1.78	98.33 ± 1.67	0.89
Isoflurane level (%)	0.69 ± 0.41	0.56 ± 0.53	0.4 ± 0.31	0.21

The data are represented as mean ± standard deviation.

* Significantly different (p < 0.05), Different superscript letters denote statistically significant differences between groups (p < 0.05)

### The puppies

Overall, 145 puppies were born. Five puppies (3.5%) were born with normal parturition and four (2.8%s) died due to dystocia before cesarean section. During cesarean section, four puppies (2.8%) were born-dead, five (3.5%) had cleft palate, and two puppies that aspirated amniotic fluid during assistance were excluded from the study. A cleft palate was observed in the French Bulldog (3/5), Bully (1/5), and Golden Retriever (1/5). The overall survival rate was 93.4% (127/136), and all born–dead puppies were delivered by emergency surgery.

A total of 125 puppies (66 females and 59 males) were included in this study. No significant differences in Apgar scores were found between the genders (p = 0.69). No significant correlation was found between the puppy’s body weight and Apgar score (r = 0.072, p = 0.16). Apgar scores significantly differed between litter numbers. Apgar scores were lower in puppies born from the first litter at 60 min compared with the second to fourth litter numbers (p = 0.03). Litter size significantly affected Apgar scores; puppies born with a litter size of 11 had significantly lower Apgar scores than those born with a litter size of one to nine (p < 0.0001). Apgar score and litter size were significantly correlated (r = –0.24, p < 0.0001) with a mild negative correlation. Ultrasound fetal heart rates were not correlated with Apgar scores (r = 0.04, p = 0.45).

Anesthetic drugs and time points after delivery had a significant effect on Apgar scores (Table-4). Apgar scores were significantly lower in the etomidate group than in the alfaxalone and propofol groups at all time points. All Apgar variables were significantly lower in the etomidate group than in the alfaxalone and propofol groups (Table-5). Heart rates in the etomidate group were significantly lower than those in the other groups at all time points (Table-6). Respiratory rates in the propofol group were significantly higher than those in the etomidate group at all time points. For viability prediction, Apgar scores were classified into three classes (Table-7). Etomidate resulted in a higher number of puppies in the low and moderate viability classes at 5 and 15 min; however, at 60 min after delivery, there were no low viability puppies. All puppies in the alfaxalone and propofol groups had high viability at 60 min after delivery.

**Table-4:** Apgar scores in puppies in all groups with different time points.

Drugs	Time point (min)			p-value (time)

	5	15	60	
Etomidate (n = 41)	6.15 ± 2.73^B,a^	6.07 ± 2.60^B,a^	7.54 ± 1.79^B,b^	<0.0001
Alfaxalone (n = 43)	8.72 ± 1.72^A,a^	9.21 ± 1.20^A,a^	9.88 ± 0.32^A,b^	0.002
Propofol (n = 41)	7.83 ± 1.92^A,a^	9.05 ± 0.84^A,b^	9.66 ± 0.66^A,c^	<0.0001
p-value (group)	<0.0001	<0.0001	<0.0001	

Data are represented as mean ± standard deviation. Difference in superscript capital letter reveals significant difference between groups (p < 0.05). Difference in superscript small letter reveals significant difference between time points (p < 0.05)

**Table-5:** Apgar scores in puppies in different groups classified by Apgar parameters.

Apgar parameters	Group	Time point (min)			p-value

		5	15	60	
Heart rate	Etomidate	0.71 ± 0.90^B,a^	0.46 ± 0.71^B,b^	0.61 ± 0.86^B,ab^	0.04
	Alfaxalone	1.65 ± 0.61^A^	1.63 ± 0.69^A^	1.88 ± 0.32^A^	0.08
	Propofol	1.37 ± 0.66^A,ab^	1.29 ± 0.68^A,a^	1.66 ± 0.66^A,b^	0.008
Mucus	Etomidate	1.39 ± 0.83^a^	1.54 ± 0.81^B,a^	2.00 ± 0.00^b^	<0.0001
	Alfaxalone	1.67 ± 0.71^a^	1.95 ± 0.30^A,b^	2.00 ± 0.00^b^	0.006
	Propofol	1.44 ± 0.87^a^	1.88 ± 0.46^A,b^	2.00 ± 0.00^b^	<0.0001
Reflex irritability	Etomidate	1.29 ± 0.81^B,a^	1.34 ± 0.73^B,a^	1.61 ± 0.49^B,b^	0.0003
	Alfaxalone	1.84 ± 0.43^A^	1.88 ± 0.39^A^	2.00 ± 0.00^A^	0.11
	Propofol	1.71 ± 0.46^A,a^	2.00 ± 0.00^A,b^	2.00 ± 0.00^A,b^	<0.0001
Motility	Etomidate	0.93 ± 0.85^B,a^	0.93 ± 0.82^B,a^	1.37 ± 0.70^B,b^	<0.0001
	Alfaxalone	1.67 ± 0.52^A,a^	1.74 ± 0.44^A,a^	2.00 ± 0.00^A,b^	0.0009
	Propofol	1.37 ± 0.73^A,a^	1.88 ± 0.33^A,b^	2.00 ± 0.00^A,b^	<0.0001
Respiratory effort	Etomidate	1.83 ± 0.44^A,ab^	1.80 ± 0.46^B,a^	1.95 ± 0.22^b^	0.02
	Alfaxalone	1.88 ± 0.32^AB^	2.00 ± 0.00^A^	2.00 ± 0.00	0.05
	Propofol	1.95 ± 0.22^B^	2.00 ± 0.00^A^	2.00 ± 0.00	0.61

Data are represented as mean ± standard deviation. Difference in superscript capital letter reveals significant difference between groups (p < 0.05). Difference in superscript small letter reveals significant difference between time points (p < 0.05)

**Table-6:** Heart rate and respiratory rate in puppies in different groups and time points.

Group	Time point (min)			p-value

	5	15	60	
Heart rate				
Etomidate	173.46 ± 55.85^B,a^	153.227 ± 48.65^B,b^	184.68 ± 40.60^B,a^	0.0001
Alfaxalone	243.7 ± 46.09^A,ab^	236.65 ± 41.08^A,a^	254.88 ± 32.03^A,b^	0.01
Propofol	220.88 ± 46.78^A,ab^	210.05 ± 34.73^A,a^	238 ± 37.26^A,b^	0.002
Respiratory rate				
Etomidate	24.76 ± 9.92^A^	25.32 ± 9.03^A^	27.93 ± 9.13^A^	0.09
Alfaxalone	28.16 ± 10.02^AB^	28.63 ± 8.78^A^	31.33 ± 7.62^A^	0.08
Propofol	30.54 ± 8.94^B,a^	34.00 ± 7.61^B,a^	38.68 ± 10.25^B,b^	<0.0001

Data are represented as mean ± standard deviation. Difference in superscript capital letter reveals significant difference between groups (p < 0.05). Difference in superscript small letter reveals significant difference between time points (p < 0.05)

**Table-7:** Apgar score classes in all groups at different time points.

Apgar score class	Etomidate (n = 41) (%)			Alfaxalone (n = 43) (%)			Propofol (n = 41) (%)		

	Time point (min)								

	5	15	60	5	15	60	5	15	60
Low (0–3)	9 (22)	6 (14.6)	0	1 (2.3)	0	0	1 (2.4)	0	0
Moderate (4–6)	13 (31.7)	17 (41.5)	15 (36.6)	3 (7)	2 (4.7)	0	9 (22)	0	0
High (7–10)	19 (46.3)	18 (43.9)	26 (63.4)	39 (91.7)	41 (95.3)	43 (100)	31 (75.6)	41 (100)	41 (100)

Data are represented as number (percentage) of the puppy

Based on type of surgery, Apgar scores in the etomidate group were significantly lower than those in the alfaxalone and propofol groups in both elective and emergency surgery (Table-8). In elective surgery, Apgar scores did not significantly differ between the anesthetic groups at 5 min after delivery. Apgar scores were significantly lower in the etomidate group than in the alfaxalone and propofol groups at 15 and 60 min after delivery. All puppies in the propofol group were in the high viability class from 15 min after delivery, which was faster than that in the alfaxalone group at 60 min after delivery. At 60 min after delivery in the etomidate group, 27.8% of the puppies were in the moderate viability class and 72.8% were in the high viability class. In emergency surgery, Apgar scores were significantly higher in the alfaxalone and propofol groups than in the etomidate group at all time points. All puppies in the alfaxalone group were classified into the high viability class at all time points. All puppies in the propofol group had high viability from 15 min after delivery. At 60 min after delivery in the etomidate group, 43.5% and 56.5% of the puppies had moderate and high viability, respectively.

**Table-8:** Apgar scores in elective and emergency surgery.

Group	Time point (min)			p-value

	5	15	60	
Elective surgery				
Etomidate (n = 18)	7.89 ± 1.88^a^	6.83 ± 2.26^B,b^	7.5 ± 1.54^B,ab^	0.004
Alfaxalone (n = 24)	8.04 ± 1.94^a^	8.88 ± 1.54^A,b^	9.79 ± 0.41^A,c^	0.0001
Propofol (n = 22)	7.59 ± 2.20^a^	9.23 ± 0.75^A,b^	9.77 ± 0.43^A,b^	<0.0001
Emergency surgery				
Etomidate (n = 23)	4.78 ± 2.54^B,a^	5.48 ± 2.74^B,a^	7.57 ± 2.00^B,b^	<0.0001
Alfaxalone (n = 19)	9.58 ± 0.84^A^	9.63 ± 0.76^A^	10.00 ± 0^A^	0.52
Propofol (n = 19)	8.11 ± 1.56^A,a^	8.84 ± 0.90^A,ab^	9.53 ± 0.84^A,b^	0.004

Data are represented as mean ± standard deviation. Difference in superscript capital letter reveals significant difference between groups (p < 0.05). Difference in superscript small letter reveals significant difference between time points (p < 0.05)

## Discussion

Alfaxalone and propofol induction, followed by isoflurane maintenance during cesarean section, were compared. Compared with propofol, induction with alfaxalone had better Apgar scores and neonatal viability during 60 min after delivery [13, 19]. In our study, Apgar scores in the alfaxalone and propofol groups were significantly higher than the etomidate group in both elective and emergency cesarean sections during 60 min after delivery. Apgar scores in the propofol group were better than the alfaxalone group in elective surgery, whereas Apgar scores in the alfaxalone group were higher than the propofol group in emergency surgery. However, there were no statistically significant differences between the two anesthetic drugs. Therefore, alfaxalone or propofol induction followed by isoflurane inhalation is recommended anesthetic protocols for canine cesarean section.

Apgar scores at the beginning of delivery are influenced by the anesthetic protocol and the scores increase with time as the anesthetic drug is metabolized and eliminated [13, 14]. In our study, at 5 min after delivery, the Apgar score in the alfaxalone group was better than that in the propofol group. This agrees with the findings of another study that alfaxalone caused less respiratory depression than propofol [20]. However, all puppies in the propofol group were in the high viability class faster than those in the alfaxalone group. Although alfaxalone and propofol have rapid onset, short duration, rapid elimination, and rapid recovery, propofol has a shorter recovery time than alfaxalone [21]. In addition, propofol has extrahepatic metabolism, which is advantageous for use in patients with limited liver and renal function and in fetuses.

Etomidate is the anesthetic induction drug of choice in dogs with preexisting cardiovascular problems because it maintains cardiovascular function [22]. However, no previous study has reported the effect of etomidate induction on Apgar scores in puppies. In this study, Apgar scores were significantly lower in the etomidate group than in the alfaxalone and propofol groups. In addition, the smooth score of anesthetic induction the etomidate group was lower than that in the alfaxalone and propofol groups. Although etomidate has a minimal effect on cardiorespiratory systems, it has its own disadvantages such as cortisol suppression, prolonged recovery, poor recovery quality, and adverse effects on the fetus [22]. Puppies have limited hepatic and renal function; therefore, the remaining plasma concentration of anesthetic drugs may reduce their vigor and viability. In addition, etomidate has more adverse effects and rougher quality during induction and recovery than alfaxalone and propofol [22]. Etomidate may be an anesthetic option for bitches with cardiovascular problems that require general anesthesia. However, Apgar score assessment is highly suggestive, and immediate neonatal assistance is needed in puppies with low viability.

Delivery time (the time from induction to the last puppy delivery) is associated with neonatal viability. All anesthetics may cross the blood-placental barrier to the fetus and cause fetal depression. Anesthetic induction drugs (injection or inhalation) with rapid distribution and redistribution allow drugs to be transferred back to the bitch. It is recommended that the delivery time should be at least 15 min before fetal delivery. No effect of a delivery time longer than 10 min has been reported previously, as well as no advantage to a short delivery time on puppy vigor, which may be caused by the high concentration of anesthetic drug remaining in the neonate’s circulation [16]. However, prolonged anesthetic duration and delivery time increase fetal hypoxia and depression. In a previous study with propofol induction and maintenance with isoflurane, a 20 min wait from induction to delivery was used, and the neonatal mortality rate was 21% [23]. In our study, the average delivery time was 6 min, with an overall survival rate of 93.4%. This agreed with Schmidt *et al*. [2] that recommended a time from induction to the start of surgery of less than 15 min. The optimal delivery time remains controversial, and further studies are required to evaluate it and improve neonatal viability in dogs.

In this study, neonatal gender and body weight had no significant effect on Apgar scores. The litter number and size may influence neonatal viability. In this study, the Apgar scores of puppies born in the first litter of the bitch were lower than those of puppies born in the second to fourth litter. In addition, the Apgar score was significantly lower in puppies born in a litter of 11 than in a litter of one to nine puppies. The litter size of seven puppies had a negative effect on the Apgar score [11], and litter size over nine puppies had a 5% increase in neonatal mortality rate [9, 24, 25]. A large litter size may limit fetal growth during pregnancy and neonatal assistance, which reduces the viability of the neonate. Therefore, it is necessary to consider the influence of the litter number of the bitch and litter size before breeding and delivery.

Of dogs with dystocia, 60% require surgical intervention [1], and brachycephalic breeds are the most common breeds [9, 14, 24]. In our study, the French Bulldog was the main breed that required cesarean section. The neonatal mortality rate in our study was 6.6% for congenital defects and born–dead puppies. In the present study, cleft palate was found mainly in French Bulldogs, which agrees with the previous studies showing that French Bulldogs have a high incidence of congenital defects and a high mortality rate [14]. In our study, all born–dead puppies underwent emergency cesarean section, which was in agreement with the previous studies that reported a high mortality rate of 19.6%–50% [2, 26]. Fetal hypoxia was the main cause of mortality, and factors associated with neonatal mortality were obstruction in the pelvic canal and anesthetic duration of more than 80 min [2, 24].

There are some limitations in this study. Although Apgar scores in the propofol group were better than the alfaxalone group in elective surgery, and Apgar scores in the alfaxalone group were higher than the propofol group in emergency surgery, the differences between these two anesthetics were not statistically significant. Therefore, increasing the number of bitches and puppies in each group and elective or emergency cesarean section may affect the statistical results. The viability of a newborn can be influenced not only by the effects of anesthesia. Evaluation of Apgar immediately after birth may be useful to reflect the initial life function of puppies. In a clinical setting, immediate neonatal care is imperative; therefore, the Apgar score at birth was not recorded in this study. Ultrasound fetal heart rates were not measured in all fetuses before cesarean section; therefore, Apgar scores were not associated. Moreover, Apgar scores can be assessed using different criteria [5, 9, 13, 14]. The results should be carefully interpreted when comparing studies using different Apgar criteria.

## Conclusion

The alfaxalone and propofol groups had better outcomes than the etomidate group with higher Apgar scores, neonatal viability, and induction quality. Therefore, alfaxalone and propofol are recommended as anesthetic induction drugs, followed by isoflurane to maintain the anesthetic stage during cesarean section.

## Authors’ contributions

TS, SJ, PW, CN, and PK: Conceived and designed the study. TS, PW, NB, and SJ: Performed the study. SS: Performed statistical analysis. TS: Analyzed and interpreted data and drafted the manuscript. TS, SJ, and PK: Revised the manuscript. All authors have read, reviewed, and approved the final manuscript.
